# Plant Protection: Focusing on Plant-Feeding Mites

**DOI:** 10.3390/plants15101534

**Published:** 2026-05-18

**Authors:** Dejan Marčić, Maria Pappas, Ismail Döker

**Affiliations:** 1 Laboratory of Applied Entomology, Institute of Pesticides and Environmental Protection, Banatska 31B, 11080 Belgrade, Serbia; 2 Laboratory of Agricultural Entomology and Zoology, Department of Agricultural Development, School of Agricultural and Forestry Sciences, Democritus University of Thrace, Pantazidou 193, 68200 Orestiada, Greece; mpappa@agro.duth.gr; 3 Laboratory of Acarology, Department of Plant Protection, Agricultural Faculty, Cukurova University, 01330 Sarıçam, Türkiye; idoker@cu.edu.tr

Higher plants provide suitable habitats for mites (Acari), the second most numerous group of arthropods. Among these, plant-feeding mites are of greatest importance as pests of agricultural crops, ornamentals and other economic plants. The most economically important pests belong to the family of spider mites (Tetranychidae), among which the two-spotted spider mite, *Tetranychus urticae* Koch, is the most notorious polyphagous and cosmopolitan pest. Additionally, economically important plant pests are also found in other mite families, such as rust and gall mites (Eriophyidae), broad mites (Tarsonemidae), and flat mites (Tenuipalpidae) [1]. Plants also host predatory mites of the family Phytoseiidae, which play a crucial ecological role as natural regulators of the abundance of tetranychids and other plant-feeding mites [2].

The use of synthetic chemical acaricides has long been the primary strategy for protecting economic plants from plant-feeding mites. However, this strategy has not proven to be sustainable in the long term, due to the rapid development and spread of acaricide resistance in spider mites, especially in *T. urticae*. Also, environmental and human health concerns have caused the withdrawal of many acaricides from the market [3,4,5]. Biological control, i.e., the use of phytoseiid predators as natural enemies of plant-feeding mites, is one of the oldest proposed solutions to address these challenges. More than 30 species of phytoseiid mites intended for augmentative biological control, based on mass release of the mites, are commercially available on the world market [6].

Various factors can significantly influence the effectiveness of phytoseiid mites as biological control agents (BCAs): host plant characteristics, environmental conditions, predator food habits, and release rates [2,7,8]. Environmental factors, such as the duration of daylight, are known to affect the predation ability of phytoseiids. Pakyari and Zemek [9] examined the influence of photoperiods of 8L:16D, 12L:12D, and 16L:8D (in hours, light:dark) on the predation rate of *Phytoseiulus persimilis* Athias-Henriot (Phytoseiidae), one of the most important BCAs, preying on *T. urticae* eggs. The daily predation rate (*Dj*), the total number of prey eggs consumed (*Pj*), the highest net predation rate (*C*_0_), the finite predation rate (ω), the transformation rate (*Qp*), and the stable predation rate (*ψ*) increased with an increase in the light photophase, reaching their peak in the 16L:8D photoperiod. Considering that, in general, the number of *T. urticae* eggs predated by *P. persimilis* was higher under longer photoperiod, the authors recommended the 16L:8D photoperiod as optimal for the biocontrol of spider mites in controlled environments [9].

Maintaining quality and reliability are important critical points in the mass production of commercialized phytoseiid species and strains [6,10]. Bechtsoudis et al. [11] evaluated the effects of rearing substrate, food type, and rearing history on the development, survival, reproduction, and predation efficiency of the predatory mite *Amblyseius andersoni* Chant (Phytoseiidae). The mites were reared on leaf discs or Plexiglas plates and fed one of five diets, including various plant pollens and the stored product mite, *Carpoglyphus lactis* (L.) (Carpoglyphidae) as a factitious prey. The substrate type did not affect development or survival of the predator contrary to the food type, with mites fed on pollen of cattail (*Typha angustifolia* L.) or *C. lactis* developing faster and producing more eggs. Survival remained high across all diets. The intrinsic rate of increase (*r*) was highest with cattail pollen and *C. lactis*. The five-generation rearing of the predator on cattail pollen did not affect its performance or feeding on natural prey such as *T. urticae* or the tomato russet mite, *Aculops lycopersici* (Tryon) (Eriophyidae). These findings demonstrate that *A. andersoni* can be effectively mass-reared on alternative diets and substrates, supporting biocontrol strategies [11].

Growing demands for environmentally friendly pest management have boosted the interest in biopesticides—pesticides of microbial or botanical origin—as an alternative to synthetic chemical pesticides. Among botanical biopesticides, products based on essential oils (EOs), volatile secondary metabolites of aromatic plants belonging to the families Lamiaceae, Myrtaceae, Rutaceae, Asteraceae and others, have recently emerged as a promising alternative to synthetic pesticides [12,13]. Research on the use of EOs as potential bioacaricides has mainly focused on *T. urticae*. Giordano et al. [14] evaluated the acaricidal effects of essential oils extracted from three Lamiaceae species: oregano (*Origanum vulgare* L.), sage (*Salvia officinalis* L.) and rosemary (*Salvia rosmarinus* Spenn.), against *A. lycopersici* under laboratory conditions. The most toxic to *A. lycopersici* was *O. vulgare* EO, causing 90% mortality at 0.5% (*w*/*v*) concentration after 4 days, while *S. rosmatinus* and *S. officinalis* EOs had more limited effects, with 46% and 42% mortality, respectively. The median lethal concentration (LC_50_) values were 2.23 mL L^–1^ for O. vulgare, 5.84 mL L^–1^ for *S. rosmarinus*, and 6.01 mL L^–1^ for *S. officinalis* EOs. These results showed that the efficacy of *O. vulgare* EO was comparable to commercially available botanical acaricides [14].

Production of the EOs and other toxic secondary metabolites is a mechanism of direct plant defence against herbivores [15]. Plants employ various direct and indirect mechanisms to defend themselves against herbivores, and many of these mechanisms are inducible. Silicon (Si) is recognized as an important element for plant nutrition and health, which induces and enhances plant defences against pests and pathogens. Hassan et al. [16] evaluated the effects of Si on life history traits and population parameters of the red spider mite, *Tetranychus macfarlanei* Baker and Pritchard (Tetranychidae), an important pest widely distributed across Tropical and Oriental regions. The mites were reared on leaf discs from country bean (*Lablab purpureus* L.) plants treated with 28 ppm and 56 ppm Si concentrations, applied as a ground drench. The higher Si concentration significantly extended total preoviposition period (TPOP) and reduced female longevity and fecundity, as well as the net reproductive rate (*R*_0_), the intrinsic rate of increase (*r*), and the finite rate of increase (*λ*). One potential mechanism behind these effects is making feeding physically difficult for mites by Si deposition [16]. A recent study [17] showed that other Si-mediated mechanisms are possible. Adding Si to the nutrient solution directly suppresses *T. urticae* population growth and reduced their damage potential to the French bean (*Phaseolus vulgaris* L.). Additionally, Si treatment promoted *P. persimilis* attraction by shifting the composition of herbivore induced plant volatiles (HIPVs) emitted in response to *T. urticae* infestation from the bean plants, as an indirect mechanism of plant defence [15,17].

Infested non-crop banker plants can induce or enhance defence mechanisms in neighbouring crop plants and alter herbivore performance by emitting HIPVs. Tasaki et al. [18] showed that HIPVs emitted from sesame (*Sesamum indicum* L.) banker plants infested with the zoophytophagous mirid bug *Nesidiocoris tenuis* (Reuter) (Hemiptera: Miridae) significantly reduced fecundity of *T. urticae* on neighbouring tomato (*Solanum lycopersicum* L.) plants, whereas those from tomato and spider flower (*Cleome hassleriana* Chodat) plants had no significant effects. Moreover, *T. urticae* infestation of banker plants suppressed mite oviposition on tomato plants. However, tomato and spider flower plants adjacent to *N. tenuis*-infested plants did not affect fecundity on the neighbouring tomato plants. These differences indicate the importance of selecting banker plant species that provide beneficial airborne cues.

In order to achieve sustainable control of spider mites and other plant-feeding mites, various alternatives to relying on the use of synthetic chemical acaricides have been considered. In addition to biological control with phytoseiid mites, the use of biological acaricides, and induction and enhancement of plant defence, various acaricide resistance management strategies have been evaluated, along with other measures. None of these control measures, however, is sustainable on its own. For modern protection of plants from plant-feeding mites, an approach that combines chemical, biological, cultural, and other control measures as an integrated pest management (IPM) programme is essential [9,14,18,19].
